# Presence of *Chlamydia trachomatis* and *Mycoplasma* spp., but not *Neisseria gonorrhoeae* and *Treponema pallidum*, in women undergoing an infertility evaluation: high prevalence of tetracycline resistance gene *tet*(M)

**DOI:** 10.1186/s13568-017-0510-2

**Published:** 2017-11-17

**Authors:** Min Li, Xiaomei Zhang, Ke Huang, Haixiang Qiu, Jilei Zhang, Yuan Kang, Chengming Wang

**Affiliations:** 1 Yangzhou University College of Veterinary Medicine, Yangzhou, Jiangsu China; 2 Northern Jiangsu People’s Hospital, Yangzhou, Jiangsu China; 3 Department of Pathobiology, College of Veterinary Medicine, Auburn University, Auburn, AL USA

**Keywords:** *Chlamydia trachomatis*, *Mycoplasma* spp., *tet*(M), *Neisseria gonorrhoeae*, *Treponema pallidum*

## Abstract

*Chlamydia trachomatis*, *Mycoplasma* spp., *Neisseria gonorrhoeae* and *Treponema pallidum* are sexually transmitted pathogens that threaten reproductive health worldwide. In this study, vaginal swabs obtained from women (n = 133) that attended an infertility clinic in China were tested with qPCRs for *C. trachomatis*, *Mycoplasma* spp., *N. gonorrhoeae*, *T. pallidum* and tetracycline resistance genes. While none of vaginal swabs were positive for *N. gonorrhoeae* and *T. pallidum*, 18.8% (25/133) of the swabs were positive for *Chlamydia* spp. and 17.3% of the swabs (23/133) were positive for *Mycoplasma* species. All swabs tested were positive for tetracycline resistance gene *tet*(M) which is the most effective antibiotic for bacterial sexually transmitted infections. The qPCRs determined that the gene copy number per swab for *tet*(M) was 7.6 times as high as that of *C. trachomatis* 23S rRNA, and 14.7 times of *Mycoplasma* spp. 16S rRNA. In China, most hospitals do not detect *C. trachomatis* and *Mycoplasma* spp. in women with sexually transmitted infections and fertility problems. This study strongly suggests that *C. trachomatis* and *Mycoplasma* spp. should be routinely tested in women with sexually transmitted infections and infertility in China, and that antimicrobial resistance of these organisms should be monitored. Further studies are warranted to determine the prevalences in different regions and associated risk factors.

## Introduction

Female infertility, including vaginal multi-pathogen infection induced infertility, is a major public health concern worldwide. *Chlamydia trachomatis*, *Mycoplasma* spp., *Neisseria gonorrhoeae* and *Treponema pallidum* have been extensively shown to be associated with infertility, particularly because of endometrial and tubal inflammation.

*Chlamydia trachomatis* is an obligate intracellular bacterial pathogen which remains the leading cause of bacterial sexually transmitted disease worldwide. Infections in the lower genital tract are frequently asymptomatic and, if untreated, can ascend to the upper genital tract, potentially leading to complications such as tubal factor infertility, and subfertility (Tang et al. 2015; Karinen et al. 2004). The organism is often undiagnosed in routine examinations performed in infertility clinical in China (Zheng et al. 2017).

*Mycoplasmas* are frequently isolated from the genital tract with *M. genitalium* and *M. hominis* considered responsible for genital diseases, infertility, and obstetric complications (Haggerty and Ness 2007; Taylor-Robinson and Lamont 2011; Kataoka et al. 2006). *M. hominis* has been found in about two-thirds of women with bacterial vaginosis and in 10% of women with salpingitis and endometritis (Judlin 2003).

*Neisseria gonorrhoeae* is the etiological agent of gonorrhea, the second most frequently reported sexually transmitted infection (STI) in the world. *N. gonorrhoeae* is a cause of pelvic inflammatory disease in women, which can lead to serious reproductive complications including tubal infertility, ectopic pregnancy, and chronic pelvic pain (Costa Lourenço et al. 2017; Kirkcaldy et al. 2016). The World Health Organization reported over 78 million new cases of gonococcal infection in people aged 15–49 worldwide during 2012 (World Health Organization 2016). Gonorrhea is usually symptomatic in men. However, there can be symptomatic gonococcal cervicitis and the complicated gonorrhea may cause infertility in women (Costa Lourenço et al. 2017; Ison 2011).

*Treponema pallidum* can lead to the complex and systemic disease, syphilis which reduces the clinical pregnancy rate after in vitro fertilization/intra-cytoplasmic sperm injection (Wang et al. 2015). Furthermore, *T. pallidum* infection with varied clinical presentations can cause neurological, cardiovascular and other multisystem damage, leading to a long time course and serious, even life-threatening consequences.

Tetracycline is the antimicrobial of choice against bacterial STIs including *C. trachomatis*, *Mycoplasmas* and *Ureaplasma*. There is a high-level of resistance to tetracyclines in genital bacteria including lactobacilli which is due mainly to the presence of the *tet*(M) gene that mediates resistance to tetracyclines (de Barbeyrac et al. 1996; Dégrange et al. 2008; Mardassi et al. 2012).

The aim of the present work was to evaluate the presence of *C. trachomatis*, *Mycoplasma* spp., *N. gonorrhoeae*, *T. pallidum* and tetracycline resistance gene in vaginal swabs of women undergoing an infertility evaluation in China.

## Materials and methods

### Vaginal samples

The protocols used in the study were approved by the Institutional Review Board of Northern Jiangsu People’s Hospital of China. In September of 2015, vaginal swabs were obtained from 133 women undergoing infertility evaluation in an infertility clinic of Northern Jiangsu Peoples Hospital, Yangzhou city, Jiangsu Province of China. Swabs were collected into sterile tubes containing 400 μl DNA/RNA stabilization buffer (Roche Molecular Biochemicals, Indianapolis, IN, USA) and stored at − 80 °C until the DNA was extracted. In addition, a questionnaire that surveyed demographic details as well as obstetric and gynecologic history was administered.

### DNA extraction

The High-Pure PCR Template Preparation Kit (Roche Molecular Biochemicals, Indianapolis, IN, USA) was used to extract total nucleic acids from vaginal swabs according to the manufacturer’s instructions and as described before (Li et al. 2016). The extracted DNA was eluted in 200 μl elution buffer.

### PCR assays

#### *Chlamydia* FRET-qPCR

The FRET-qPCR used in this study followed the protocols described by DeGraves et al. (2003) and Guo et al. (2016) and was performed in a LightCycler 480-II real-time PCR platform. This PCR assay targets 168-bp fragment of the *Chlamydia* spp. 23S rRNA gene, and could detect all 11 *Chlamydia* species with a detection sensitivity of single copy/reaction. The PCR products were further verified by electrophoresis through 2% agarose gels (BIOWEST^®^, Hong Kong, China), purification using the QIAquick PCR Purification Kit (Qiagen), and sequencing with forward and reverse primers (BGI, Shanghai, China).

#### *Mycoplasma* FRET-qPCR

To investigate the presence of *Mycoplasma* in the vaginal swabs, a set of primers and probes were designed using Vector NTI to amplify a 174-bp fragment of the *Mycoplasma* spp. 16S rRNA gene (Table 1). PCR amplification was performed in a LightCycler 480-II real-time PCR platform using a high-stringency 18-cycle step-down temperature protocol: 6 × 1 s @ 95 °C, 12 s @64 °C, 8 s @ 72 °C; 9 × 1 s @ 95 °C, 12 s @ 62 °C, 8 s @ 72 °C; 3 × 1 s @ 95 °C, 12 s @ 60 °C, 8 s @ 72 °C; followed by 30 low-stringency cycles: 30 × 1 s @ 95 °C, 12 s @ 57 °C, 30 s @ 67 °C, and 10 s @ 72 °C. Twenty μl PCR reactions were prepared containing 10.0 μl DNA template, 0.2 μl forward primer (100 μM), 0.2 μl reverse primer (100 μM,), 4.0 μl 5× PCR buffer, 0.4 μl 10 μM dNTP, 0.3 μl 5 U/μl *Taq* DNA polymerase and 4.9 μl ultrapure H_2_O. The PCR products were further verified, purified, and sequenced as mentioned above. A standard PCR with a long amplicon (703–713 bp) of the 16S rRNA gene were performed on positive samples based on FRET-PCR to determine the *Mycoplasma* species (Yoshida et al. 2002).

**Table 1:** Oligonucleotide primers used in this study

PCR	Target	Primer/probe	Sequence (5′–3′)	Size (bp)	Ref
Generic for 11 *Chlamydia* species	23S rRNA	UP1	GGGGTTGTAGGRTTGRGGAWAAAGGATC	168	Guo et al. (2016)
		UP2	GGGGTTGTAGGGTCGATAAYATGRGATC		
		DN	GAGAGTGGTCTCCCCAGATTCARACTA		
		FLU1	ACGAAAGGAGAKMAAGACYGACCTCAAC-6-FAM		
		FLU2	ACGAAAAAACAAGAGACTCTATTCGAT-6-FAM		
		LCRed	LCRed640-CCTGAGTAGRRCTAGACACGTGAAAC-P		
Generic for *Mycoplasma* species	16S rRNA	UP	CTGCCTGAGTAGTAYRYTCGCAA	174	This study
		DN	TGCACCATCTGTCACTHBGTTARCCTC		
		FLU	AAACCACATGCTCCACCGCTTGT-36-FAM		
		LCRed	LCRed640-GGTCCCCGTCAATTCCTTTAAGTTT-P		
*Mycoplasma* species for sequencing	16S rRNA	UP	ACTCCTACGGGAGGCAGCAGTA	700	Yoshida et al. (2002)
		DN	TGCACCATCTGTCACTCTGTTAACCTC		
*N. gonorrhoeae*	porA pseudogene	UP	CGGTTTCCGTGCGTTACGA	132	Whiley et al. (2004)
		DN	AACTGGTTTCATCTGATTACTTTCCA		
		FLU	CATTCAATTTGTTCCGAGTCAAAACAGC-6-FAM		
		LCRed	LCRed640-AGTCCGCCTATACGCCTGCTACTTTCAC-P		
*T. pallidum*	polA	UP	GGTAGAAGGGAGGGCTAGTA	104	Heymans et al. (2010)
		DN	CTAAGATCTCTATTTTCTATAGGTATGG		
		TaqMan probe	FAM-ACACAGCACTCGTCTTCAACTCC-BHQ1		
RPPs gene		UP	CCACCGAATCCTTTCTGGGC	245	This study
		DN	ATCCGAAAATCTGCTGGGGTACT		

For use as quantitative standards, the products of the *Mycoplasma* FRET-PCR on vaginal swabs were gel purified using a QIAquick Gel Extraction Kit (Qiagen, Valencia, CA, USA). An aliquot of the purified product was sequenced for confirmation at GenScript (Nanjing Jiangsu, China) and the remainder quantified (ng/ml) with the Quanti-iT™ PicoGreen^®^ dsDNA Assay Kit. After using the molecular mass of the 16S rRNA gene to calculate the molarity of the solution, dilutions were made to give solutions containing 10,000, 1000, 100, 10, and 1 gene copies per reaction. These were amplified by *Mycoplasma* FRET-PCR in triplicate to determine the detection limit of the PCR.

The specificity of this PCR was further verified with the amplification of DNAs s from *Salmonella* Typhimurium (ATCC 14028), *Escherichia coli* (ATCC25922), *C. trachomatis* (ATCC VR-571B), *Ehrlichia canis*, *Anaplasma phagocytophilum* and *Rickettsia felis*.

#### *Neisseria gonorrhoeae* and *T. pallidum* qPCR

Two sets of primers were used to detect *N. gonorrhoeae* and *T. pallidum* by the *porA* pseudogene and the *polA* gene respectively as described (Whiley et al. 2004; Heymans et al. 2010).

#### RPPs gene qPCR

A qPCR was established to quantify a class of tetracycline resistance genes encoding for ribosomal protection proteins (RPPs) in vaginal swabs in this study. The *tet*(M), *tet*(S), *tet*(O), *tet*(Q), *tet*(T), *tet*(36), *tet*(44) sequences were obtained from NCBI (http://www.ncbi.nlm.nih.gov). Using the Clustal Multiple Alignment Algorithm, we identified a highly conserved 245-bp PCR target on the above seven tetracycline resistance genes (Table 1). The thermal protocol was identical to what described above for the *Mycoplasma*-qPCR. The specificity of the primers was verified by BLASTn and DNA sequencing of obtained PCR products. The sensitivity of the *tet*(M)-qPCR was determined as mentioned above.

### Statistical analysis

All statistical analyses were performed with the Statistica 7.0 software package (StatSoft, Inc., Oklahoma, USA). Positivity of pathogens in different age groups was compared using the Chi squared Test. Differences of copy numbers of pathogens and *tet*(M) gene in different age groups were logarithmically transformed and analyzed with the two-tailed Tukey honest significant difference (HSD) test in one-way ANOVA. Differences at P ≤ 0.05 were considered significant.

## Results

### Establishment of the *Mycoplasma* FRET qPCR and *tet*(M) qPCR

The *Mycoplasma* FRET qPCR established in this study detected the target *Mycoplasma* 16S rRNA with a detection limit of one copy 16S rRNA per reaction. It did not detect *Salmonella* Typhimurium, *Escherichia coli*, *C. trachomatis*, *Ehrlichia canis*, *Anaplasma phagocytophilum* and *Rickettsia felis.* The RPPs gene-qPCR we established had a detection limit of one gene copy per reaction while its specificity was verified by sequencing.

### Prevalence and copy numbers of *C. trachomatis*, *Mycoplasma* spp., *N. gonorrhoeae*, *T. pallidum* and *tet*(M) in vaginal swabs from infertile women

While none of the swabs were positive for *N. gonorrhoeae* and *T. pallidum*, 31.6% of the swabs (42/133) were positive for *C. trachomatis* and/or *Mycoplasmas* infection. In six women (4.5%) coinfection with both *C. trachomatis* and *Mycoplasma* spp. was observed. All the swabs were positive for the *tet*(M) gene.

*C. trachomatis* was found to be the only chlamydial species in all vaginal swabs with a positivity of 18.8% (25/133). The average gene copy number of *C. trachomatis* was 27,855 (± 21,301 SEM) per swab.

**Fig. 1:**
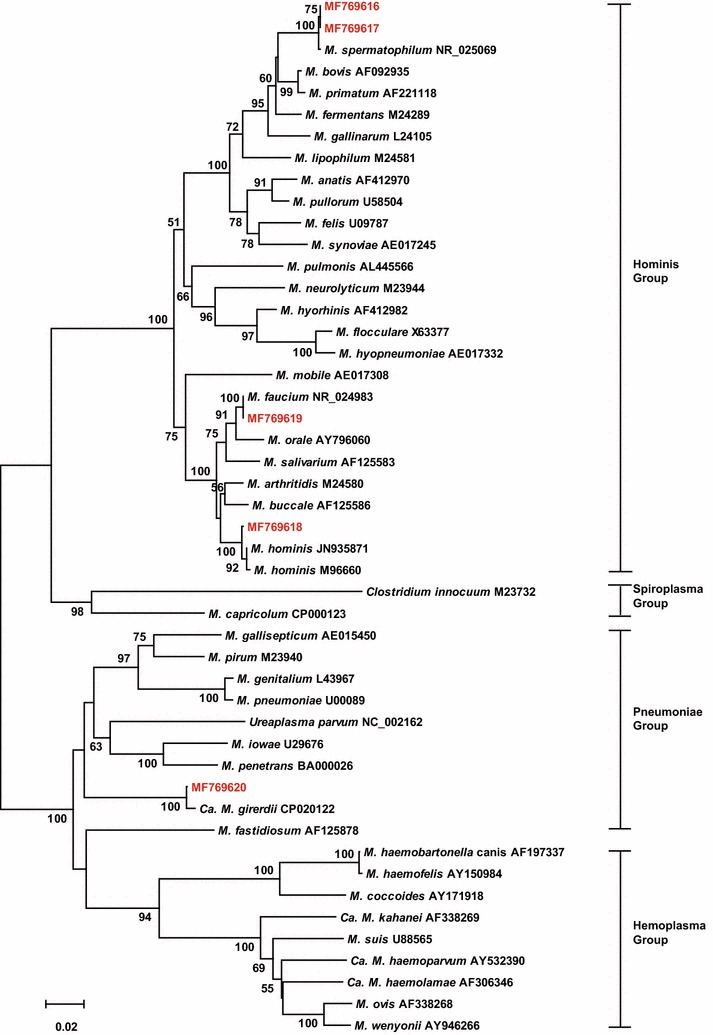
Neighbor-joining phylogenetic tree based on the sequence alignment of the 16S rRNA gene (700 bp). The five strains identified in our study (MF769616–MF769620) are in red font. The sequence identified in our study (MF769616 and MF769617) are most similar to *M. spermatophilum*, MF769618 is most similar to *M. hominis*, MF769619 is most similar to *M. faucium*, MF769620 is most similar to *Ca. M. girerdii*. Bootstrap percentage values greater than 50% are given at the nodes of the tree (1000 replicates)

The FRET-qPCR followed by standard PCR that generates a longer amplicon of the 16S rRNA gene determined that 17.3% (23/133) of swabs were positive for *Mycoplasma* spp. Sequence analysis of these amplicons revealed that *M. spermatophilum* (MF769616, n = 2; MF769617, n = 2), *M. hominis* (MF769618, n = 14), *M. faucium* (MF769619, n = 4), and *Candidatus Mycoplasma girerdii* (*Ca. M. girerdii*) (MF769620, n = 1) (Fig. 1). The average gene copy number for *Mycoplasma* spp. was 14,433 (± 7872 SEM) per swab.

**Fig. 2:**
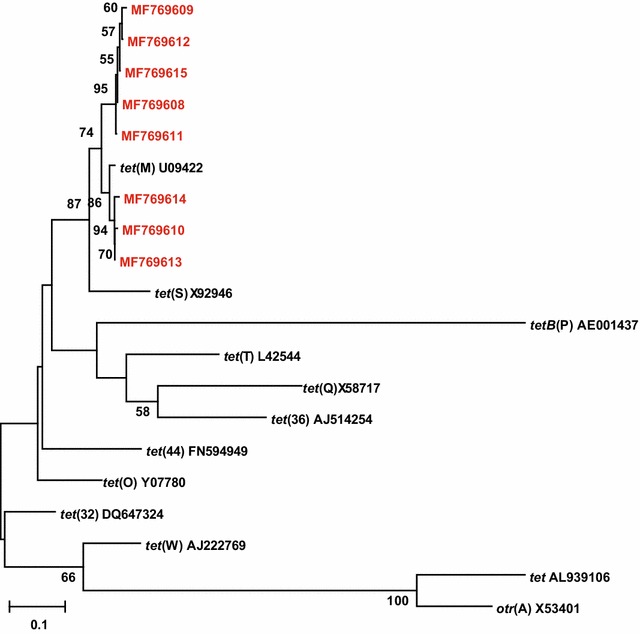
Neighbor-joining phylogenetic tree based on sequences of the RPPs gene (245 bp). The tetracycline resistance genes identified in our study (MF769608–MF769615, in red font) are the most similar to *tet*(M). Bootstrap percentage values greater than 50% are given at the nodes of the tree (1000 replicates)

Sequence analysis of 22 RPPs PCR products revealed eight different *tet*(M) sequences (MF769608–MF769615) (Fig. 2). The sequences of MF769608 and MF769620 in this study were identical to the *tet*(M) sequences (KU545550 and MF422120) in GenBank.

All the vaginal swabs were positive for *tet*(M) with the average gene copy number per swabs being 212,878 (± 39,025 SEM) which was 7.6 times that for *C. trachomatis* 23S rRNA and 14.7 times that for *Mycoplasma* spp. 16S rRNA.

### Prevalence and copy numbers of *C. trachomatis*, *Mycoplasma* and *tet*(M) in different age groups

The average age of the 128 women for whom we had data was 29.35 years (range 21–44 years). For analysis we divided the women into four groups: 21–25, 26–31, 32–37, and 38–44 years.

**Fig. 3:**
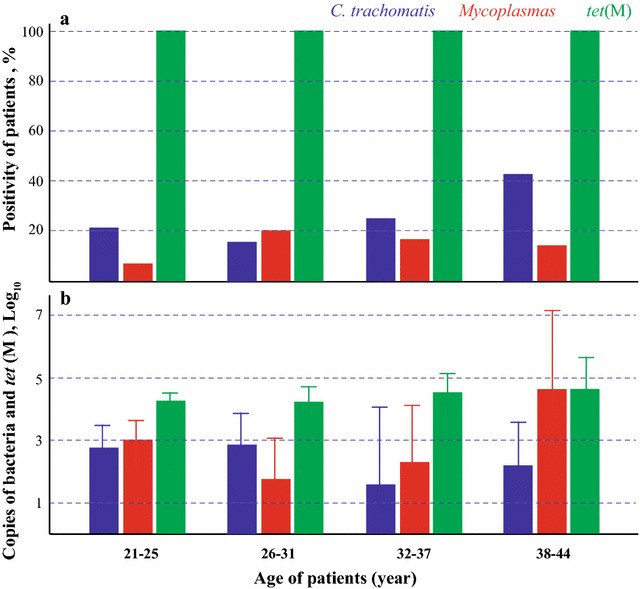
Prevalence and copy numbers of *C. trachomatis*, *Mycoplasma* and *tet*(M) gene in vaginal samples in four age groups. **a** The *C. trachomatis* positivity in 21–25 age group, 26–31 age group, 32–37 age group and 38–44 age group detected in this study were 21.4, 15.7, 25.0 and 42.9% respectively. The *Mycoplasma* positivity in above four age groups detected in this study were 7.1, 20.5, 16.7 and 14.3% respectively. The *tet*(M) gene positivity in above four age groups were all 100%.** b** The average gene copy numbers for *C. trachomatis* 23S rRNA, *Mycoplasma* spp. 16S rRNA and *tet*(M) gene were 10^2.53 ± 1.05 SEM^, 10^2.81 ± 1.24 SEM^, 10^4.28 ± 1.34 SEM^ (being equivalent to 27,855 ± 21,301 SEM; 14,433 ± 7872 SEM; 212,878 ± 39,025 SEM), respectively. The gene copy number for four age groups did not differ significantly for *C. trachomatis*, *Mycoplasma* spp. and *tet*(M). However, the *tet*(M) copy number is significantly higher than *C. trachomatis* (averagely 7.6 times), and *Mycoplasma* spp. (averagely 14.7 times)

The positivity for *C. trachomatis* in the 38–44 age group (42.9%) was significantly higher (P < 0.01) than in the younger age groups (32–37 years, 25.0%; 21–25 age group (21.4%) and 26–31 age group (15.7%) (Fig. 3). Similarly, the positivity for *Mycoplasma* in the 21–25 age group (7.1%) was significant lower (P < 0.01) than in the older age groups (26–31 age group, 20.5%; 32–37 age group, 16.7%; 38–44 age group, 14.3%) (Fig. 3).

The average gene copy numbers of *C. trachomatis*, *Mycoplasma* and *tet*(M) did not differ significantly between the four age groups (Fig. 3) although the total gene copy number per swab was significantly higher for *tet*(M) (P < 10^−4^) than for *C. trachomatis* 23S rRNA and *Mycoplasma* spp. 16S rRNA.

## Discussion

This study demonstrated that 31.6% of women attending an infertility clinic in China tested positive for *C. trachomatis* and/or *Mycoplasma* spp. whereas these two pathogens are usually not detected in women with STIs and fertility problems in most Chinese hospitals (Peng et al. 2017; Bao et al. 2016). In contrast, we failed to identify the two pathogens (*N. gonorrhoeae* and *T. pallidum*) that are at the top of the list for STI surveillance in China. Surprisingly, all the swabs tested in this study were positive for *tet*(M), the resistance gene against tetracycline which is the most commonly used and most effective antimicrobial for bacterial STIs. Our study strongly suggests that *C. trachomatis* and *Mycoplasma* spp. should be routinely tested for in women with STIs and infertility in China, and that the antimicrobial resistance of these organisms should be monitored.

The Chinese government launched a massive campaign to eliminate STIs in the 1950s, and STIs were thought to be extremely uncommon by the 1960s in China (Cohen et al. 1997). However, since the early 1980s, STIs have reemerged with the introduction of the open door policy and economic liberalization and are now recognized as a major public-health problem in China as they have spread from the high risk group to the general population (Chen et al. 2007).

In China, HIV/AIDS, syphilis, and gonorrhea are reportable STIs according to the Law of the People’s Republic of China on Prevention and Treatment of Infectious Diseases. The reported incidence of primary and secondary syphilis was 11.7 cases per 100,000 residents in 2009, an increase of 2.1-fold since 2005 (Chen et al. 2011). However, the reported incidence of gonorrhea has decreased by about 30% (Chen et al. 2011) with the overall prevalence of *N. gonorrhoeae* infection in the general population being 0.08% for women and 0.02% for men (Parish et al. 2003). The overall trend is that the prevalence of *N. gonorrhoeae* and *T. pallidum* has dropped significantly in China (Chen et al. 2011; Adachi et al. 2016), congruent to the findings as none of the 133 vaginal swabs were tested positive for *N. gonorrhoeae* and *T. pallidum* in this study.

*C. trachomatis* infections of the lower female genital tract are frequently asymptomatic. However, if infections do not resolve or persist untreated, organisms can ascend and pathology in the upper genital tract, potentially causing salpingitis and functional damage to the fallopian tubes and tubal factor infertility (Hafner 2015). The incidence of genital *C. trachomatis* infections increased by nearly 40% from 37.20 per 100,000 persons in 2010 and 51.3 per 100,000 in 2014 in Guangdong Province of China (Wong et al. 2017). Based on a Chinese Health and Family Life Survey, the prevalence of *C. trachomatis* was 2.6% (95% CI 1.6–4.1%) in Chinese women (Conejero et al. 2013; Parish et al. 2003). This is similar to the 2.7% (n = 1717) positivity for women reported in the Netherlands (Morré et al. 1999). While the international standards recommend annual screening for *C. trachomatis* in sexually active women (Conejero et al. 2013), most hospitals in China do not detect *C. trachomatis* in women during regular checks or in women with STIs and fertility problems. The high prevalence of *C. trachomatis* demonstrated in this study strongly suggests that hospitals in China should follow the international recommendation and national guideline for routine surveillance of *C. trachomatis*.

*Mycoplasma* species have been reported to be associated with perinatal morbidity and mortality while *M. hominis* has been mostly associated with chorioamnionitis and thought to be an etiological agent (Taylor-Robinson and Lamont 2011). The overall prevalence of urogenital *Mycoplasma* infections varies in different countries while international reports suggest an increase in infections due to *Mycoplasma* over the last decade (Díaz et al. 2013). The global prevalence of *M. genitalium* among symptomatic and asymptomatic sexually active women ranges between 1 and 6.4% (Pereboom et al. 2014). This variability in prevalence rates reported in different countries is perhaps due to differences in detection methods, types of samples studied, sample sizes, hygiene issues, socioeconomic status, age of participants, and absence of regular screening, treatment, and control programs (Ahmadi et al. 2016). This study identified 22/23 of *Mycoplasmas* sequences being *hominis* taxa, and only one sequence was identified as *Ca. M. girerdii*.

For decades, tetracyclines, as broad-spectrum antibiotics, have been used extensively for treating bacteria-induced STIs. Resistance to tetracyclines is now increasingly prevalent in STIs, and the drugs usefulness in treating urogenital infections is decreasing because of the presence of the *tet*(M). Surprisingly, in our study 100% of the swabs we tested were positive for *tet*(M) gene which was present in very high copy numbers, 7.6 times that of *C. trachomatis* 23S rRNA and 14.7 times that of *Mycoplasma* spp. 16S rRNA. This indicates the *C. trachomatis* and *Mycoplasma* spp. carried multiple copies of the *tet*(M) or that the gene was also present in other vaginal microbes such as lactobacilli. The antimicrobial resistance features of vaginal bacteria should be regularly monitored to provide the judicious treatment of STIs. The phylogenetic analysis based on eight sequences identified in this study and 12 species RPPs gene in GenBank revealed that all eight sequences belong to *tet*(M) taxa, which is one of the most prevalent class of RPPs gene conferring antibiotic resistance.

Traditionally, the diagnosis of genitourinary pathogens has been based on bacterial culture. Bacterial isolation, however, is cumbersome, costly, and time-consuming. It is also selective in that samples must be appropriately collected, transported and stored to maximize the number of viable bacteria. It is particularly difficult to grow obligate intracellular bacterial such as *C. trachomatis* while cell lines or chicken embryos are required for their growth. In this study, sensitive and specific qPCRs were applied for direct testing of samples for pathogens and resistant genes. This approach avoids the limitations of bacterial isolation and the resultant underestimation of pathogen prevalence.

In conclusion, we found a high prevalence of *C. trachomatis*, *Mycoplasma* spp. and *tet*(M) in vaginal swabs from women with fertility problems in China, whilst *N. gonorrhoeae* or *T. pallidum* were not detected. While more studies are warranted to investigate the prevalences of these organisms and *tet*(M) in larger populations from different regions, our data should alert health workers in China that *C. trachomatis* and *Mycoplasma* spp. might be the main pathogens in women with STIs and infertility problem.

## Abbreviations

*tet*: tetracycline resistance gene
FRET-qPCR: fluorescence resonance energy transfer quantitative PCR
STI: sexually transmitted infection
RPPs: ribosomal protection proteins
